# HIV risk in an urban American population

**DOI:** 10.1186/1742-4690-9-S1-P99

**Published:** 2012-05-25

**Authors:** Josephine F Wilson

**Affiliations:** 1 Boonshoft School of Medicine, Wright State University, Kettering Oh, USA

## Introduction

Investigations of specialized populations in the United States have revealed multiple risk behaviors for HIV infections. Identification of significant risk factors for specific populations enables tailoring interventions to populations. The present study examined risk behaviors in a general urban population in order to determine the need for HIV interventions in subgroups of the general population.

## Materials and methods

HIV testing tied to a small remuneration was offered to adults in a small city in Ohio. Testing was conducted in churches, public health sites, and in a van that was parked at various public housing sites. A total of 3,290 individuals were tested for HIV. At the time of testing, demographic and risk behavior data were collected. Data were analyzed using logistic regression to determine the populations most at risk for HIV infection in this large sample of poor urban residents.

## Results

The sample tested ranged in age from 18 to 85 years, was 80.2% Black and 18.8% Caucasian, and 43.6% male. Altogether, 0.4% of the sample tested was HIV positive. In this largely African American sample, 77.3% admitted to condom use, 4.9% had sex with an intravenous drug user (IDU), 4.2% used intravenous drugs, 2.1% had sex with a MSM, and 11.4% had sex with 3 or more partners in the past 30 days. Logistic regression indicated significant associations between a positive HIV test and having sex with a MSM, no condom use, being an IDU, having sex with an IDU, and having sex with someone who is HIV positive. Odd ratios were calculated for each of the identified significant risk factors.

## Conclusions

These data indicate where intervention efforts are needed in our community. Studies of this sort enable public health administrators to conserve valuable prevention funds by targeting interventions to populations at greatest risk. This study revealed that, in this largely African American population, men and women were equally at risk for HIV, thus mandating the development of HIV interventions for men and women in this urban population.

